# Neuron-specific enolase as a prognostic biomarker in acute ischemic stroke patients treated with reperfusion therapies

**DOI:** 10.3389/fneur.2024.1408111

**Published:** 2024-07-18

**Authors:** Tiago Esteves Freitas, Ana Isabel Costa, Leonor Neves, Carolina Barros, Mariana Martins, Pedro Freitas, Duarte Noronha, Patrício Freitas, Teresa Faria, Sofia Borges, Sónia Freitas, Eva Henriques, Ana Célia Sousa

**Affiliations:** ^1^Stroke Centre, Hospital Dr. Nélio Mendonça, Funchal, Portugal; ^2^Internal Medicine Department, Hospital Dr. Nélio Mendonça, Funchal, Portugal; ^3^Internal Medicine Department II, Hospital Prof. Doutor Fernando Fonseca, Amadora, Portugal; ^4^Neurology Department, Hospital Dr. Nélio Mendonça, Funchal, Portugal; ^5^Centro de Investigação Clínica Dra. Maria Isabel Mendonça, Funchal, Portugal

**Keywords:** ischemic stroke, biomarkers, neuron specific enolase, reperfusion therapies, functional outcome, prognosis, neurological disability

## Abstract

**Introduction:**

Ischemic stroke is a significant global health concern, with reperfusion therapies playing a vital role in patient management. Neuron-specific enolase (NSE) has been suggested as a potential biomarker for assessing stroke severity and prognosis, however, the role of NSE in predicting long-term outcomes in patients undergoing reperfusion therapies is still scarce.

**Aim:**

To investigate the association between serum NSE levels at admission and 48 h after reperfusion therapies, and functional outcomes at 90 days in ischemic stroke patients.

**Methods:**

This study conducted a prospective cross-sectional analysis on consecutive acute ischemic stroke patients undergoing intravenous fibrinolysis and/or endovascular thrombectomy. Functional outcomes were assessed using the modified Rankin Scale (mRS) at 90 days post-stroke and two groups were defined according to having unfavorable (mRS3-6) or favorable (mRS0-2) outcome. Demographic, clinical, radiological, and laboratory data were collected, including NSE levels at admission and 48 h. Spearman’s coefficient evaluated the correlation between analyzed variables. Logistic regression analysis was performed to verify which variables were independently associated with unfavorable outcome. Two ROC curves determined the cut-off points for NSE at admission and 48 h, being compared by Delong test.

**Results:**

Analysis of 79 patients undergoing reperfusion treatment following acute stroke revealed that patients with mRS 3–6 had higher NIHSS at admission (*p* < 0.0001), higher NIHSS at 24 h (*p* < 0.0001), and higher NSE levels at 48 h (*p* = 0.008) when compared to those with mRS 0–2. Optimal cut-off values for NSE_0_ (>14.2 ng/mL) and NSE_48h_ (>26.3 ng/mL) were identified, showing associations with worse clinical outcomes. Adjusted analyses demonstrated that patients with NSE_48h_ > 26.3 ng/mL had a 13.5 times higher risk of unfavorable outcome, while each unit increase in NIHSS_24h_ score was associated with a 22% increase in unfavorable outcome. Receiver operating characteristic analysis indicated similar predictive abilities of NSE levels at admission and 48 h (*p* = 0.298). Additionally, a strong positive correlation was observed between NSE_48h_ levels and mRS at 90 days (*r* = 0.400 and *p* < 0.0001), suggesting that higher NSE levels indicate worse neurological disability post-stroke.

**Conclusion:**

Serum NSE levels at 48 h post-reperfusion therapies are associated with functional outcomes in ischemic stroke patients, serving as potential tool for patient long-term prognosis.

## 1 Introduction

Prognostication plays an essential role in decision making by offering patients and their families the necessary information to set realistic and achievable care goals. It helps determine eligibility for specific benefits and targets interventions to those who are most likely to benefit. Prognostication includes three main components: clinicians estimate the likelihood of a particular outcome over a certain period using their clinical judgment or other tools; this estimate is shared with the patient according to their preferred way of receiving information; and the patient or their surrogate uses this information to make informed clinical decisions (1).

Age, stroke severity, stoke mechanism, infarct location, comorbid conditions, clinical findings, and related complications influence stroke prognosis. Whether reperfusion therapies (fibrinolysis and/or thrombectomy) are conducted and the quality of stroke unit care given, including early start of physical rehabilitation and prompt introduction of secondary prevention measures, also influence the outcome of ischemic stroke (2).

Patient history, clinical examination and imaging technology have been the cornerstone in assessing stroke patients, guiding therapeutic decisions, and monitoring disease progression. Akin to the established gold standard in Cardiology, where biomarkers such as high sensitivity troponins have revolutionized the assessment of myocardial infarction (3), incorporating biomarkers into stroke management holds immense potential. These biomarkers offer a promising avenue to identify patients at heightened risk of severe disease, tailor treatment strategies, predict the response to reperfusion therapies and reasonably predict the overall prognosis and outcomes. While numerous proteins serve as markers of brain tissue damage, inflammation and coagulation/thrombosis, their utility is hindered by a lack of specificity to ischemic stroke, given that various other diseases processes can also damage the brain tissue (4).

For a biomarker to prove useful in stroke management, it should meet some basic criteria: be specific to the brain tissue, rise immediately within hours of a tissue insult, proportionally reflect the extent of brain damage, and ultimately serve as a reliable prognostic indicator for the event (5). Numerous biomarkers have been associated with short-and long-term clinical outcomes after stroke, but most of them have failed to improve the prediction capacities of conventional clinical variables. Biomarkers of ischemic brain injury include S100 calcium binding protein B (S-100B), neuron-specific enolase (NSE), myelin basic protein and glial fibrillary acid protein, among others (6).

The NSE is a dimeric intracellular neuronal glycolytic enzyme, primarily located in the cytoplasm of neurons, cells of the diffuse neuroendocrine system and erythrocytes. Elevated levels of NSE have been observed in response to sudden central nervous system events, such as cerebral infarction, subarachnoid hemorrhage, head injury, hypoxia, seizures, and cardiac arrest. These conditions are characterized by the disruption of the blood–brain barrier and subsequent damage of neuronal cells, leading to a leakage of NSE, which can be detected in cerebral spinal fluid but also in saliva or blood samples (7). Several studies propose NSE as a marker of brain damage following an ischemic event. There is also a correlation between NSE levels and acute ischemic stroke severity, infarction volume, extent of brain tissue damage [as clinically measured by the National Institutes of Health Stroke Scale (NIHSS) score], and poor functional outcomes (5, 8, 9).

NSE levels change dynamically following symptom onset, reflecting the necrosis of neuronal cells within the ischemic penumbra. Lower NSE levels are associated with clinical diffusion mismatch (7), serving as an alternative indicator for salvageable ischemic tissue that may be more responsive to interventions such as intravenous fibrinolysis and/or mechanical thrombectomy. However, high NSE levels are not exclusive to ischemic stroke and can also be found in other diseases such as neuroendocrine cell cancers like small cell lung cancers, neuroblastomas, melanomas and carcinoid tumors (10).

Since its approval by the European Stroke Organization in 2008, intravenous thrombolysis with alteplase has been a recognized systemic reperfusion treatment for patients with acute ischemic stroke (11). The pivotal MR CLEAN study published in 2015 further solidified the landscape by demonstrating the safety, efficacy and favorable impact on functional outcomes associated with intraarterial thrombectomy (12).

Building upon these advancements, our study aimed to evaluate the relationship between NSE levels at patient admission and 48 h post-reperfusion therapies, and functional outcome in ischemic stroke patients.

## 2 Materials and methods

### 2.1 Participants

This was a prospective cross-sectional study targeting consecutive patients with acute ischemic stroke admitted do the Stroke Centre of Hospital Dr. Nélio Mendonça between March 1st to October 31st of 2023 and treated with recombinant tissue type plasminogen activator (rtPA) within 4.5 h after symptom onset and/or endovascular treatment with thrombectomy up to 24 h after symptom onset. Endovascular treatment was indicated in patients with large vessel occlusion after discussion with the interventional neuroradiology team. Stroke onset was defined as the last time the patient was seen well, without any neurological deficit. Inclusion and exclusion criteria for intravenous rtPA were used in accordance with those used in the ECASS III (13). Patients eligible for thrombolysis received 0.9 mg of alteplase (Actilyse^®^, Boehringer Inglheim, Ingelheim am Rhein, Germany) per kilogram, administered intravenously (with an upper limit of 90 mg).

Patients eligible for thrombectomy were generally treated with a guide-catheter (Infinity Plus^®^ by Stryker, Neuronmax^®^ by Penumbra or Cerebase^®^ by Cerenovus), an aspiration catheter (Catalyst^®^ 5, 6 or 7 or Vecta 74^®^ by Stryker, ACE68^®^ or 4MAX^®^ by Penumbra or Embovac^®^ by Cerenovus) and a microcatheter (Trevo Trak^®^ or AXS Offset^®^ by Stryker, 3MAX^®^ by Penumbra or Prowler^®^ by Cerenovus), guided by a microwire (Synchro-14^®^ by Stryker or Neuroscout^®^ by Cerenovus); whenever deemed necessary by the interventionalist, a stent retriever was also used (Trevo NXT^®^ by Stryker or Embotrap^®^ by Cerenovus).

Informed consent was obtained from patients or their family members to participate in the study. The study protocol was approved by the local ethics committee.

### 2.2 Measures

#### 2.2.1 Demographic and medical history

Upon arrival to the emergency department, all patients were evaluated by the on-duty stroke physician. Standard neurological examinations, electrocardiogram, chest radiography, bloodwork and computed tomography angiography (CTA) scans of the brain, cerebral arteries, cervical arteries, and aortic arch where performed. The following clinical data were collected: (1) patient age and gender; (2) degree of neurological deficit determined by the NIHSS score at hospital admission and 24 h after reperfusion therapies; (3) risk factors of stroke including history of hypertension (HTN), diabetes mellitus (DM), hyperlipidemia, current or former smoking (14), atrial fibrillation (AF) (15), heart failure (HF) (16), previous stroke (17), coronary artery disease (CAD) and previous myocardial infarction (18); and (4) prior medical treatments (anti-hypertensives, anti-diabetics, antiplatelets, direct oral anticoagulants, warfarin and statins).

#### 2.2.2 Clinical assessment

Stroke severity was assessed by a NIHSS score (19) certified stroke physician, and was categorized as mild (0–4 points), moderate (5–15 points) and severe (16–42 points). Furthermore, stroke was classified according to the Bamford/Oxfordshire Community Stroke Project Classification criteria (20) in: total anterior circulation infarction (TACI), partial anterior circulation infarction (PACI), posterior circulation infarction (POCI) and lacunar infarction (LACI).

Clinical outcomes at 90 days were also assessed by certified investigators using the mRS (21) in a follow-up appointment. Outcomes were dichotomized as favorable (mRS score of 0–2 points) and unfavorable (mRS score of 3–6 points). All patients underwent a CT scan at 24 h post-reperfusion therapies or whenever neurological deterioration occurred to assess for the presence of ICH. CT scans were reviewed by an experienced neuroradiologist who was blinded to clinical details and laboratory data. Symptomatic intracranial hemorrhage (sICH) was defined as any extravascular blood within the brain or the cranium that was associated with clinical deterioration, defined by a rise of at least 4 points in the NIHSS score and/or leading to death, and that was considered to be the predominant cause for neurologic deterioration. Complications during the stroke unit stay, including pneumonia, urinary tract infections, sepsis, pulmonary embolism, deep venous thrombosis, and pressure sore ulcers, were recorded (22).

#### 2.2.3 Radiological assessment

All patients underwent a CT and CTA scans of the brain, cerebral arteries, cervical arteries and aortic arch at admission and a brain CT scan at 24 h after reperfusion therapies.

#### 2.2.4 Laboratory test

All patients had baseline blood samples drawn in the emergency room to determine: levels of NSE, glucose, HbA_1_C, hemoglobin and CRP. NSE levels were measured again in every patient at 48 h post-reperfusion therapies. Blood samples obtained were collected in chemistry test tubes. After centrifugation, serum samples were separated, and kept frozen at −80°C until assayed. NSE analysis was performed using Cobas^®^ e801 (Roche) and the reference values were 0–17 ng/mL. Glucose and CRP were assessed using Cobas^®^ c702 (Roche). HbA_1_C analysis was performed by a D-100^®^ system (Biorad). Hemoglobin was assessed using a DxH 800^®^ hematology analyzer (Beckman Coulter). Since NSE is also present in erythrocytes, hemolyzed samples were discarded. All laboratory analyses were carried out in the Clinical Pathology laboratory of the Hospital Dr. Nélio Mendonça with quality accreditation, from the national and official model of the Portuguese Ministry of Health, based on the Agencia de Calidad Sanitaria de Andalucia (ACSA) Model (international version).

#### 2.2.5 Statistical analysis

Continuous variables were described as mean ± SD or median (minimum-maximum) and compared with Student t-test or Mann–Whitney U-test, as appropriate. The number of patients and percentages for categorical variables were given, and compared using a X^2^ or Fisher exact test, as appropriate. The Spearman coefficient was applied to verify the correlation between examined variables. The relative risks of each variable for an unfavorable outcome were estimated as odds ratios (OR) in a logistic regression analysis.

A receiver-operating characteristic (ROC) curve in conjunction with the Youden’s index were applied to determine the cut-off of NSE, at admission and at 48 h post-reperfusion therapies, that distinguished between favorable and unfavorable outcome, and compared by Delong test.

SPSS 25 software (IBM SPSS Statistic) was used to perform statistical analysis.

A level of *p* < 0.05 was accepted as statistically significant.

## 3 Results

A total of 85 consecutive patients who fulfilled the established criteria for reperfusion treatment (either thrombolysis, thrombectomy or both) were included in the study. Of these patients six were excluded: two patients due to follow-up loss at 90 days post-stroke, three due to hemolyzed blood samples that invalidated the NSE level result, and one due to lung cancer history. Consequently, 79 patients (50.6% female; mean age 69.2 ± 14.4 years) were enrolled into the present study. The median time from symptom onset to hospital door was 113 min and the median time from symptom to reperfusion treatment as 165 min (when patients underwent both treatments, the time of rtPA bolus was chosen as the reperfusion treatment time, as it was the first treatment applied). The median NIHSS score was 10 points (range 0 to 30) before, and 8 points (range 0 to 32) 24 h after reperfusion therapies. The median NSE level at admission (NSE_0_) was 16.3 ng/mL (4.7–45.1) and the median NSE level at 48 h post-reperfusion therapies (NSE_48h_) was 17.5 ng/mL (4.7–117.0). At 90 days post-stroke, patients were assessed and distributed into two groups according to the mRS score at that time: 44 (55.7%) were placed in the favorable outcome group with a mRS score of 0–2 points, and 35 (44.3%) were in placed in the unfavorable outcome group with a mRS score of 3–6 points. Both groups were compared regarding baseline characteristics as shown in Table 1. There were no significant differences between the two groups in terms of sex, history of DM, hyperlipidemia, AF, previous stroke, smoking, CAD, time from symptom onset to hospital-door, time from symptom onset to treatment, fibrinolysis or thrombectomy and ICH. However, compared to favorable outcome group, patients in the unfavorable outcome group were more likely to be older (*p* = 0.006), have history of HTN (*p* = 0.012), HF (*p* = 0.019), and history of previous medical treatment (*p* = 0.029). They also presented a higher NIHSS score both at admission (*p* < 0.0001) and at 24 h (*p* < 0.0001) post-reperfusion therapies. Lastly, they were also more likely to have suffered a TACI type-stroke (*p* < 0.0001), post-stroke medical complications (*p* < 0.0001) and sICH (*p* = 0.006) (Table 1).

**Table 1:** Baseline characteristics of patients with and without unfavorable outcome (mRS 3–6) at 90 days.

Characteristics	Total (*n* = 79)	mRS (0–2) (*n* = 44)	mRS (3–6) (*n* = 35)	*p*-value
Age, years	69.2 ± 14.4	65.3 ± 15.0	74.0 ± 12.0	**0.006**
Female, *n* (%)	40 (50.6)	23 (52.3)	17 (48.6)	0.744
History of hypertension, *n* (%)	44 (55.7)	19 (43.2)	25 (71.4)	**0.012**
History of diabetes mellitus, *n* (%)	19 (24.1)	10 (22.7)	9 (25.7)	0.758
History of dyslipidemia, *n* (%)	36 (45.6)	16 (36.4)	20 (57.1)	0.065
Previous stroke, *n* (%)	18 (22.8)	12 (27.3)	6 (17.1)	0.286
Smokers, *n* (%)	20 (25.3)	10 (22.7)	10 (28.6)	0.553
History of atrial fibrillation, *n* (%)	7 (8.9)	3 (6.8)	4 (11.4)	0.693
History of heart failure, *n* (%)	8 (10.1)	1 (2.3)	7 (20.0)	**0.019**
History of coronary artery disease, *n* (%)	6 (7.6)	1 (2.3)	6 (14.3)	0.083
History of previous medical treatment, *n* (%)	53 (67.1)	25 (56.8)	28 (80.0)	**0.029**
NIHSS_0_ score	10.0 (0.0–30.0)	7.0 (0.0–21.0)	17.0 (4.0–30.0)	**<0.0001**
NIHSS_24h_ after reperfusion treatment	8.0 (0.0–32.0)	3.0 (0.0–21.0)	18.0 (1.0–32.0)	**<0.0001**
Time from symptom onset to hospital-door (min)	113.0 (10.0–720.0)	99.5 (10.0–720.0)	130.0 (15.0–720.0)	0.914
Time from symptom onset to treatment (min)	165.0 (35.0–900.0)	155.0 (35.0–900.0)	170.0 (45.0–860.0)	0.611
Oxfordshire classification – TACI, *n* (%)	45 (57.0)	15 (34.1)	30 (85.7)	**<0.0001**
Fibrinolysis or Thrombectomy, *n* (%)	59 (74.7)	35 (79.5)	24 (68.6)	0.265
Post stroke medical complications, *n* (%)	39 (49.4)	9 (20.5)	30 (85.7)	**<0.0001**
Intracranial hemorrhage (ICH), *n* (%)	14 (17.7)	6 (13.6)	8 (22.9)	0.286
Symptomatic intracranial hemorrhage (sICH), *n* (%)	6 (7.6)	0 (0.0)	6 (17.1)	**0.006**

Notes – values are presented as mean ± SD or median (minimum-maximum) for continuous variables, and number (percentages) for categorical variables. NIHSS, National Institutes of Health Stroke Scale; TACI, total anterior circulation infarct; ICH, intracranial hemorrhage; sICH, symptomatic intracranial hemorrhage; mRS, modified Rankin Scale. Statistical significance (*p* < 0.05) is represented by bold values.

Of the 14 patients with ICH, 8 were asymptomatic, 6 had sICH and 1 died of sICH within 7 days after treatment. Among the 13 deceased patients, 4 died due to malignant infarct, 4 due to nosocomial pneumonia, 3 due to sepsis (urinary tract and endocarditis primary infections), 1 due to sICH and 1 due to hemorrhagic shock (gastric ulcer).

### 3.1 Biomarkers and clinical outcome

The biomarkers and hematologic parameters in the two groups are shown in Table 2. CRP median levels were significantly higher in the unfavorable outcome group (57.4 vs. 5.5; *p* < 0.0001), as were NSE levels at 48 h (NSE_48h_) (21.0 vs. 15.5; *p* = 0.008). The NSE median levels at admission (NSE_0_) were higher in the unfavorable outcome group (19.0 vs. 15.4), without statistical significance (*p* = 0.059).

**Table 2:** Biomarkers of patients with and without unfavorable outcome (mRS 3–6) at 90 days.

Biomarkers	Total (*n* = 79)	mRS (0–2) (*n* = 44)	mRS (3–6) (*n* = 35)	*p*-value
Hemoglobin	12.6 (8.7–17.0)	12.9 (8.7–17.0)	5.8 (4.9–8.0)	0.077
HbA1C (%)	5.7 (4.4–9.2)	5.6 (4.4–9.2)	11.9 (9.2–16.7)	0.098
Glycemia	129.0 (88.0–259.0)	130.0 (92–259)	125.0 (88.0–194.0)	0.564
CRP	13.2 (0.6–419.0)	5.5 (0.6–419.0)	57.4 (1.0–279.0)	**<0.0001**
NSE_0_ (ng/mL)	16.3 (4.7–45.1)	15.4 (5.7–45.1)	19.0 (4.7–39.8)	0.059
NSE_48h_ (ng/mL)	17.5 (4.7–117.0)	15.5 (4.7–63.2)	21.0 (8.4–117.0)	**0.008**

Notes – values are presented as median (minimum-maximum). HbA1C, hemoglobin A1C; CRP, C reactive protein and NSE; neuron-specific enolase; mRS, modified Rankin Scale. Statistical significance (*p* < 0.05) is represented by bold values.

### 3.2 Comparison of baseline characteristics, biomarker NSE_0_ and NSE_48h_ and clinical outcome

The optimal cut-off value to distinguish unfavorable from favorable outcomes was calculated for both NSE_0_ and NSE_48h_, using a receiver operating characteristics (ROC) curve in conjunction with the Youden’s index: NSE_0_ was 14.2 ng/mL, with sensitivity of 80% and specificity of 45.5%; and NSE_48h_ was 26.3 ng/mL with sensitivity of 43.3% and specificity of 97.7%. To further estimate the clinical importance of the NSE level, all patients were divided into two subgroups according to the cut-off value of NSE_0_ and NSE_48_. Low and high NSE_0_ levels were defined as: ≤14.2 ng/mL and > 14.2 ng/mL, respectively; and NSE_48h_ levels as: ≤ 26.3 ng/mL and > 26.3 ng/mL, respectively. Baseline characteristics and clinical outcomes classified according to NSE_0_ and NSE_48h_ subgroups are shown in Tables 3, 4.

**Table 3:** Baseline characteristics and clinical outcome in patients classified according to NSE at admission (NSE_0_) subgroups.

Characteristics	NSE_0_ ≤ 14.2 (*n* = 27)	NSE_0_ > 14.2 (*n* = 52)	*p*-value
Age, years	67.7 ± 14.7	69.9 ± 14.3	0.529
Female, *n* (%)	13 (48.1)	27 (51.9)	0.750
History of hypertension, *n* (%)	12 (44.4)	32 (61.5)	0.161
History of diabetes mellitus, *n* (%)	5 (18.5)	14 (26.9)	0.407
History of hyperlipidemia, *n* (%)	10 (37.0)	26 (50.0)	0.273
History of previous stroke, *n* (%)	7 (25.9)	11 (21.2)	0.631
History of smoking, *n* (%)	11 (40.7)	9 (17.3)	**0.031**
History of atrial fibrillation, *n* (%)	1 (3.7)	6 (11.5)	0.412
History of heart failure, *n* (%)	2 (7.4)	6 (11.5)	0.564
History of coronary artery disease, *n* (%)	2 (7.4)	4 (7.7)	1.000
History of previous medical therapy, *n* (%)	14 (51.9)	39 (75.0)	**0.038**
NIHSS_0_	13.0 (2.0–24.0)	10.0 (0.0–30.0)	0.860
NIHSS_24h_	6.0 (0.0–31.0)	10.0 (0.0–32.0)	0.313
Oxfordshire classification – TACI, *n* (%)	13 (48.1)	32 (61.5)	0.254
Thrombolysis or Thrombectomy, *n* (%)	22 (81.5)	37 (71.2)	0.317
Unfavorable outcome 90d – mRS (3–6)	7 (25.9)	28 (53.8)	**0.018**
Post stroke medical complications, *n* (%)	11 (40.7)	28 (53.8)	0.269
Intracranial hemorrhage (ICH), *n* (%)	4 (14.8)	10 (19.2)	0.626
Symptomatic intracranial hemorrhage (sICH), *n* (%)	3 (11.1)	3 (5.8)	0.406
Mortality, *n* (%)	2 (7.4)	10 (19.2)	0.165
Hemoglobin (g/dL)	12.7 (8.7–16.7)	12.5 (8.8–17.0)	0.549
HbA1C (%)	5.6 (4.6–7.4)	5.7 (4.4–9.2)	0.149
CRP (mg/dL)	8.0 (0.6–419.0)	19.7 (0.6–302.0)	0.114

Notes – values are presented as mean ± SD or median (minimum-maximum) for continuous variables, and number (percentages) for categorical variables. NIHSS, National Institutes of Health Stroke; TACI, total anterior circulation infarction; mRS, modified Rankin Scale; HbA1C, hemoglobin A1C; CRP, C reactive protein and NSE, neuron-specific enolase. Statistical significance (*p* < 0.05) is represented by bold values.

**Table 4:** Baseline characteristics and clinical outcome in patients classified according to NSE at 48 h (NSE_48h_) subgroups.

Characteristics	NSE_48h_ ≤ 26.3 (*n* = 59)	NSE_48h_ > 26.3 (*n* = 14)	*p*-value
Age, years	68.1 ± 14.5	74.1 ± 15.1	0.172
Female, *n* (%)	30 (50.8)	6 (42.9)	0.591
History of hypertension, *n* (%)	32 (54.2)	10 (71.4)	0.242
History of diabetes mellitus, *n* (%)	15 (25.4)	3 (21.4)	0.755
History of hyperlipidemia, *n* (%)	25 (42.4)	8 (57.1)	0.318
History of previous stroke, *n* (%)	16 (27.1)	1 (7.1)	0.112
History of smoking, *n* (%)	15 (25.4)	3 (21.4)	0.755
History of atrial fibrillation, *n* (%)	5 (8.5)	0 (0.0)	0.576
History of heart failure, *n* (%)	3 (5.1)	4 (28.6)	**0.007**
History of coronary artery disease, *n* (%)	2 (3.4)	4 (28.6)	**0.011**
History of previous medical therapy, *n* (%)	39 (66.1)	11 (78.6)	0.367
NIHSS_0_	9.0 (0.0–24.0)	17.5 (6.0–30.0)	**0.004**
NIHSS_24h_	6.0 (0.0–30.0)	19.5 (3.0–32.0)	**<0.0001**
Oxfordshire classification – TACI, *n* (%)	28 (47.5)	13 (92.9)	**0.002**
Thrombolysis or Thrombectomy, *n* (%)	46 (78.0)	8 (57.1)	0.110
Unfavorable outcome 90d – mRS (3–6)	17 (28.8)	13 (92.9)	**<0.0001**
Post stroke medical complications, *n* (%)	21 (35.6)	13 (92.9)	**<0.0001**
Intracranial hemorrhage (ICH), *n* (%)	10 (16.9)	4 (28.6)	0.321
Symptomatic intracranial hemorrhage (sICH), *n* (%)	3 (5.1)	3 (21.4)	0.080
Mortality, *n* (%)	4 (6.8)	6 (42.9)	**<0.0001**
Hemoglobin (g/dL)	12.8 (8.7–12.8)	11.4 (8.8–14.4)	0.053
HbA1C (%)	5.6 (4.4–9.2)	5.8 (4.7–6.4)	0.661
CRP (mg/dL)	7.4 (0.6–419.0)	116.5 (3.4–302.0)	**<0.0001**

Notes – values are presented as mean ± SD or median (minimum-maximum) for continuous variables, and number (percentages) for categorical variables. NIHSS, National Institutes of Health Stroke; TACI, total anterior circulation infarction; mRS, modified Rankin Scale; HbA1C, hemoglobin A1C; CRP, C reactive protein and NSE, neuron-specific enolase. Statistical significance (*p* < 0.05) is represented by bold values.

Regarding the NSE_0_ group, there were no significant differences in terms of sex, history of HTN, DM, hyperlipidemia, previous stroke, HF, AF, NIHSS_0_ and NIHSS_24_. Patients in the high NSE_0_ subgroup had a higher percentage of non-smokers (*p* = 0.031) and history of previous medication intake (*p* = 0.038). They also had more frequent unfavorable outcomes (*p* = 0.018) than those in the low NSE_0_ subgroup (Table 3).

Concerning the NSE_48h_ group, there were no significant differences in terms of sex, history of HTN, DM, hyperlipidemia, previous stroke, smoking and previous medication intake. Patients in the high NSE_48h_ group were more likely to have HF (*p* = 0.007) and history of CAD (*p* = 0.011). High NSE_48h_ subgroup was associated with higher NIHSS at admission (NIHSS_0_) and NIHSS at 24 h (NIHSS_24h_) scores (*p* = 0.004 and *p* < 0.0001). They also had higher percentage of unfavorable outcomes (*p* < 0.0001), TACI type-strokes (*p* = 0.002), post-stroke medical complications (*p* < 0.0001), and higher CRP median levels (*p* < 0.0001) than those in the low NSE_48h_ subgroup. Mortality was also more frequent among the high NSE_48_ subgroup (*p* < 0.0001) (Table 4).

### 3.3 Unfavorable outcome at 90 days in reperfused patients

After multivariate analysis, the variables that were independently and significantly associated with unfavorable outcome were NIHSS_24h_ score and NSE_48h_.

Patients with NSE_48h_ > 26.3 ng/mL have a 13.5 higher risk of having an unfavorable outcome (95% CI 1.2–150.1, *p* = 0.035). As the NIHSS_24h_ increases, the risk of having an unfavorable outcome increases by 22% (95% CI 1.1–1.4, *p* < 0.0001) (Table 5).

**Table 5:** Relative risks for unfavorable outcome (mRS 3–6) at 90 days in patients with reperfusion treatment.

Variables	B	S.E.	Wald	df	OR 95% CI	*p*-value
NIHSS_24h_	0.200	0.056	12.616	1	1.221 (1.094–1.363)	**<0.0001**
NSE_48h_ > 26.3 ng/mL	2.599	1.231	4.461	1	13.456 (1.206–150.145)	**0.035**

Notes – variables that did not remain in the equation: hyperlipidemia, heart failure, hypertension, NIHSS at admission, TACI type stroke, hemoglobin, hemoglobin A1C, post stroke medical complications, NSE > 14.2 ng/mL. B – Beta coefficient; S.E. – Standard error; df – degrees of freedom; OR – Odds ratio; CI, confidence interval; NIHSS – National Institutes of Health Stroke; NSE – Neuron-specific enolase; statistical significance (*p* < 0.05) is represented by bold values.

### 3.4 Receiver operating characteristic analysis of NSE at the different sampling times

**Figure 1:**
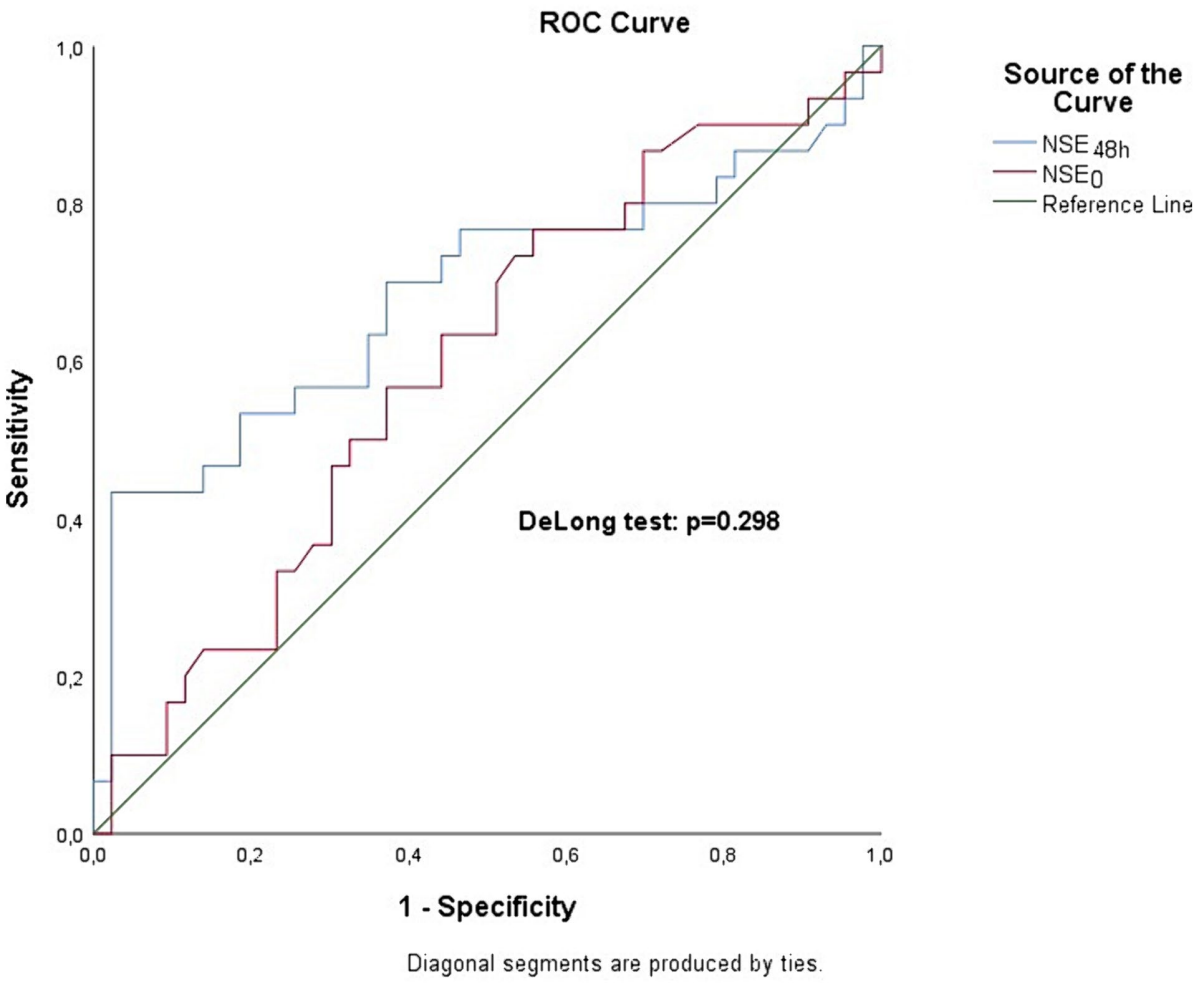
Receiver operating characteristics (ROC) analysis of NSE_0_ and NSE_48h_. The area under the curve (AUC) is 0.597 and 0.683 at admission and 48 h, respectively. There are no significant differences between AUCs, *p* = 0.298.

By comparing the two ROC curves of NSE_0_ and NSE_48h_ through the Delong test, there were no significant differences between the two curves (*p* = 0.298) regarding unfavorable outcome (Figure 1). The area under curve at admission and 48 h are similar (0.597 and 0.683, respectively).

### 3.5 Correlation between NSE_48h_ and mRS at 90 days

**Figure 2:**
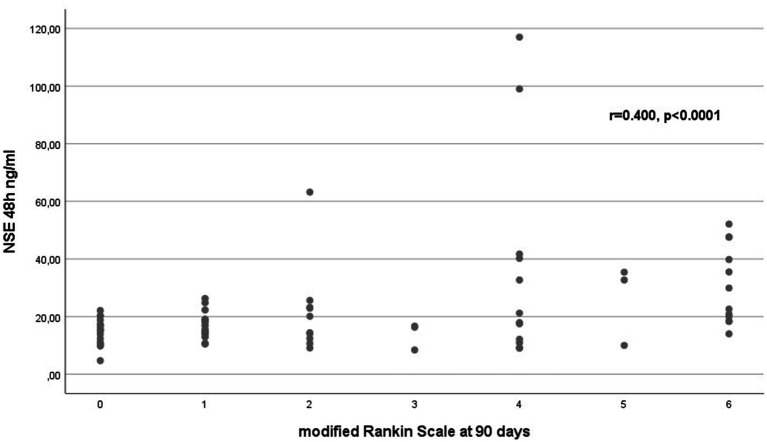
Spearman correlation between NSE_48h_ and modified Rankin Scale at 90 days. R coefficient is positive and significant (*r* = 0.400; *p* < 0.0001).

We found highly significant positive correlation between NSE_48h_ levels and mRS at 90 days (*r* = 0.400 and *p* < 0.0001) (Figure 2). The graph demonstrates that patients with an adverse neurological disability (higher mRS) had a significant higher release of the marker.

## 4 Discussion

This study evaluated the predictive value of neuron-specific enolase (NSE) levels in determining stroke outcomes for patients undergoing reperfusion therapies. It was found that older age, history of hypertension (HTN), heart failure (HF), and previous medication use were significantly associated with unfavorable outcomes (mRS score of 3–6). Higher NIHSS scores at baseline and 24 h post-reperfusion were linked to worse outcomes and increased mortality. Notably, the study revealed that higher NSE levels at 48 h post-stroke were significantly associated with more severe outcomes and greater neurological disability, while NSE levels at admission showed a non-significant trend towards worse outcomes.

Clinicians are often expected to predict outcome after stroke, whether by patient, family, or other healthcare workers. There are a wide variety of factors that influence stroke prognosis, including age, stroke severity, stroke mechanism, infarct location, reperfusion treatments, comorbid conditions, neurological disability, and stroke complications (23, 24).

The modified Rankin Scale has been used as a measure of stroke-related handicap and is frequently used as a global measure of the functional impact of stroke. In addition, the mRS at 90 days after intravenous thrombolysis or endovascular interventions for acute ischemic stroke is the proposed “core metric” of most interventional trials and worldwide comprehensive stroke centers (25). The mRS score shows moderate correlation with the volume of cerebral infarction (26).

NSE has predictive value for determining severity and early neurobehavioral outcomes after stroke (9). However, the role of NSE in predicting long-term outcomes is still an evolving area, and evidence regarding its role in ischemic stroke patients undergoing reperfusion therapies is still scarce.

In this study we demonstrated that risk factors such as older age, a history of HTN, HF and previous medication were associated with unfavorable outcomes (mRS score of 3–6 points). Previous studies (27–29) have shown a clear associated between age, history of HTN and HF with stroke prognosis. In our study, hyperlipidemia and CAD showed a non-significant trend to be more prevalent among patients with unfavorable outcomes. Recent study by Chang et al. (30) showed a poorer outcome in stroke patients with CAD. A study from Menet et al. (31), mention that hyperlipidemia exacerbate vascular damage and is importantly associated to an increased risk of mortality in stroke patients.

According to Muir et al. (32), the NIHSS score offers the best specificity, sensitivity, and accuracy for predicting prognosis, with high scores correlating proportionally with higher mortality rates (33). As was demonstrated by Chalos study et al. (34), our study showed that higher NIHSS_0_ and NIHSS_24_ post-reperfusion therapies are associated with unfavorable outcome and higher mortality rates.

In Yang’s study (35), TACI type-strokes exhibited worse 3-month clinical outcomes and higher mortality rates. Similarly, in our study, patients with ischemic stroke undergoing reperfusion therapies, we found that the TACI type-stroke was associated with stroke severity, unfavorable outcomes (85.7%) and mortality (100%) when compared to non-TACI type-strokes (PACI, LACI and POCI).

Bustamante et al. (36) reported that post-stroke complications significantly impact stroke outcomes. In our study, stroke complications were statistically significantly associated with unfavorable outcomes (*p* < 0.0001). Thirty-nine patients (49.4%) experienced post-stroke complications, with pneumonia and urinary tract infection being the most frequent. Although infections commonly occur after stroke and are strongly associated with an unfavorable outcome in these patients, effective management strategies for post-stroke infection remain scarce, presenting a significant challenge for stroke care (37).

Our study demonstrated that the median values of the biomarker CRP at admission were significantly higher among patients with unfavorable outcomes. Two studies (38, 39) indicate a strong association of poor outcomes with high sensitivity (hs)-CRP measurements at 24–48 h and 7 days post-stroke, reflecting impairment of the recovery process due to prolonged inflammation after ischemic stroke. Similar associations were also observed with CRP values at admission (40).

Since 2005 (41), we have known that NSE levels are higher in stroke patients than in controls. However, it was after Zaheer et al. (42) that a correlation was made between NSE levels on day 1, infarct volume and functional outcomes at 30 days post-stroke. Consistent with this, previous studies focusing on stroke patients not-submitted to reperfusion treatments suggested that the initial NSE level positively associated with the degree of neurological deficit (41). One single study (43) reported that NSE values did not differ between patients who underwent intravenous thrombolysis alone versus conservative medical treatment.

In our study, the NSE_48h_ levels were statistically significantly associated with an unfavorable outcome and the NSE_0_ levels were non-significantly higher in the unfavorable outcome group. Wunderlich et al. (44) reported that the release pattern of NSE rises 2–3 h after stroke onset, then decreases until 12 h, followed by a secondary increase until the last measurement on day 5 after stroke onset, probably reflecting secondary mechanism of brain damage, ongoing neuronal cell death or persistent disturbance of the blood–brain barrier. NSE levels have been shown to change dynamically after an ischemic stroke. A pathological neurovascular status on admission resulted in a significantly higher release of NSE from 48 h onward, although NSE concentrations from 18 h onward were highly correlated with the severity of the corresponding neurological deficit as quantified by the NIHSS. Considering that reperfusion therapies may increase the odds of vessel recanalization rates, possibly interfering with NSE levels, we measured the NSE level at admission (immediately before reperfusion therapies), and at 48 h post-reperfusion therapies.

A previous study (45) showed that the NSE level at 24 h post-intravenous fibrinolysis highly correlated with the severity of the corresponding neurological deficit as quantified by the NIHSS score at 24 h post-fibrinolysis.

There are several possible explanations for this finding, but the explanation that garners the most consensus is related with the penumbra area involved in the ischemic stroke. Cells in the ischemic penumbra, as they suffer necrosis, release NSE that passes from the cerebral spinal fluid to the peripheral blood through an impaired blood–brain barrier (46). Patients submitted to thrombolysis show decreased NIHSS scores as the occluded artery recanalizes and the ischemic penumbra area reperfuses, leading to a decrease in NSE levels. However, no studies have been conducted with ischemic stroke patients submitted to thrombectomy. On the other hand, patients who poorly respond to thrombolysis (considered as no or subpar recanalization) have larger core and smaller penumbra areas, leading to a higher NSE release from disrupted neuronal cells and both higher serum NSE levels and NIHSS scores.

In our study, higher NSE_0_ levels showed a non-significant trend toward an unfavorable outcome (*p* = 0.059), reflecting the above correlation between NSE and the penumbra area (42). By the other hand, higher concentration of NSE_48h_ were statistically significantly associated with an unfavorable outcome (*p* = 0.008), reflecting a larger area of necrosis and infarct core and consequently higher neurological disability.

Two previous studies (42–45) showed a similar NSE level threshold for poor functional outcomes. As mentioned before, low NSE levels might be associated with clinical-diffusion mismatch, which has been a surrogate for brain tissue at risk of infarct.

By comparing the two ROC curves of NSE_0_ and NSE_48h_ through the DeLong test, we found no significant differences between the two curves, leading us to conclude that the NSE_0_ and NSE_48h_ have identical predictive value for unfavorable outcome.

Our study shown that NSE_48h_ and NIHSS_24h_ were independently and significantly associated with unfavorable outcome, after multivariate analysis adjusted for other risk factors for unfavorable outcome.

A previous study (9) showed that the NSE levels had high predictive value for the degree of short-term disability (measured with NIHSS at day 7). In the current study the NSE_48h_ levels positively correlated with the severity of neurological long-term disability (mRS at 90d), reflecting the NSE utility as a marker for destructive process in the central nervous system, a marker of neuron loss and consequently associated with higher disability.

This study has some limitations that must be considered. First, our data result from hyperacute ischemic stroke treated with reperfusion therapies, without a control group. Whether reperfusion treatments interfere with the blood–brain barrier, increasing the serum NSE concentration is still unknown. Thus, the association between NSE, especially at 48 h, and stroke severity estimated by the NIHSS score may have been overestimated. Second, a small sample size might have confounded the results of factors predicting the severity and outcome of stroke and resulted in wider confidence intervals. The authors are still recruiting more patients to increase the sample size and further ascertain this hypothesis. We also note that the titration of NSE levels is time consuming and vulnerable to the collecting methods, resulting in hemolysis and making the interpretation of hemolytic samples impossible. Eventually, in the future, a saliva NSE sample test could be more feasible, safe, and economic (47).

In conclusion, our results show that NSE levels at 48 h are associated with patient’s deficits assessed by the mRS. Like NIHSS score, NSE can be used, on a large scale in clinical practice, as a valuable tool in clinical management and prognostic estimation in ischemic stroke patients undergoing reperfusion therapies.

In the future, NSE levels might also be used in combination with CT perfusion of brain to study the core and penumbra area, maximizing the sensitivity of both.

## Data availability statement

The raw data supporting the conclusions of this article will be made available by the authors, without undue reservation.

## Ethics statement

The studies involving humans were approved by Hospital Ethics Committee, Hospital Dr. Nélio Mendonça. The studies were conducted in accordance with the local legislation and institutional requirements. The participants provided their written informed consent to participate in this study.

## Author contributions

TiF: Writing – original draft, Writing – review & editing. AC: Writing – review & editing. LN: Writing – review & editing. CB: Writing – review & editing. MM: Writing – review & editing. PeF: Writing – review & editing. DN: Writing – review & editing. PaF: Visualization, Writing – review & editing. TeF: Writing – review & editing, Visualization. SB: Writing – review & editing. SF: Writing – review & editing, Data curation, Methodology. EH: Writing – review & editing. AS: Writing – review & editing, Writing – original draft.
